# The effects of probiotics administration on the gut microbiome in adolescents with anorexia nervosa—A study protocol for a longitudinal, double‐blind, randomized, placebo‐controlled trial

**DOI:** 10.1002/erv.2876

**Published:** 2021-11-30

**Authors:** Eva‐Maria Gröbner, Michael Zeiler, Florian Ph. S. Fischmeister, Kathrin Kollndorfer, Sonja Schmelz, Andrea Schneider, Nina Haid‐Stecher, Kathrin Sevecke, Gudrun Wagner, Lara Keller, Roger Adan, Unna Danner, Annemarie van Elburg, Benny van der Vijgh, Karlijn Liselotte Kooij, Serguei Fetissov, Nadia A. Andreani, John F. Baines, Astrid Dempfle, Jochen Seitz, Beate Herpertz‐Dahlmann, Andreas Karwautz

**Affiliations:** ^1^ Eating Disorders Unit at the Department of Child and Adolescent Psychiatry Medical University of Vienna Vienna Austria; ^2^ Institute of Psychology University of Graz Graz Austria; ^3^ Department of Biomedical Imaging and Image‐guided Therapy Medical University of Vienna Vienna Austria; ^4^ Department of Child and Adolescent Psychiatry Medical University of Innsbruck Innsbruck Austria; ^5^ Department of Child and Adolescent Psychiatry Psychosomatics and Psychotherapy RWTH Aachen University Aachen Germany; ^6^ Department of Translational Neuroscience University Medical Center Utrecht Utrecht The Netherlands; ^7^ Altrecht Eating Disorders Rintveld Zeist The Netherlands; ^8^ Faculty of Sciences INSERM UMR University of Rouen Mont‐Saint‐Aignan France; ^9^ Institute for Experimental Medicine Max Planck Institute for Evolutionary Biology Kiel University Plön Germany; ^10^ Institute of Medical Informatics and Statistics Christian Albrecht‐University Kiel Kiel Germany

**Keywords:** anorexia nervosa, gut microbiome, gut–brain axis, probiotics, randomized controlled trial

## Abstract

**Objective:**

Knowledge on gut–brain interaction might help to develop new therapies for patients with anorexia nervosa (AN), as severe starvation‐induced changes of the microbiome (MI) do not normalise with weight gain. We examine the effects of probiotics supplementation on the gut MI in patients with AN.

**Method:**

This is a study protocol for a two‐centre double‐blind randomized‐controlled trial comparing the clinical efficacy of multistrain probiotic administration in addition to treatment‐as‐usual compared to placebo in 60 patients with AN (13–19 years). Moreover, 60 sex‐ and age‐matched healthy controls are included in order to record development‐related changes. Assessments are conducted at baseline, discharge, 6 and 12 months after baseline. Assessments include measures of body mass index, psychopathology (including eating‐disorder‐related psychopathology, depression and anxiety), neuropsychological measures, serum and stool analyses. We hypothesise that probiotic administration will have positive effects on the gut microbiota and the treatment of AN by improvement of weight gain, gastrointestinal complaints and psychopathology, and reduction of inflammatory processes compared to placebo.

**Conclusions:**

If probiotics could help to normalise the MI composition, reduce inflammation and gastrointestinal discomfort and increase body weight, its administration would be a readily applicable additional component of multi‐modal AN treatment.

## Key points

- Patients with anorexia nervosa face profound alterations of the gut microbiome which does not normalise with weight gain.
- Alterations in the gut microbiome in patients with anorexia nervosa are linked to psychopathological symptoms and neurophysiological deficits, for example, related to the reward system.
- This is the first study examining the effects of probiotics administration in adolescents with anorexia nervosa using a randomized controlled trial design.

**Abbreviations**
AN: anorexia nervosa
BMI: body mass index
DSM‐5: Diagnostic and Statistical Manuel Of Mental Disorders—5th version
ICD‐10: International Classification of Diseases and Related Health Problems—10th revision
ITT: intention to treat
MI: microbiome
PUFA: poly‐unsaturated fatty acids

## 1 INTRODUCTION

Anorexia nervosa (AN) is a complex and serious mental disorder characterised by a distorted body image, fear of gaining weight, extreme dietary restriction accompanied by severe weight loss and psychiatric comorbidities (American Psychiatric Association, 2013). A recently published meta‐analysis show that eating disorders including AN have a high prevalence worldwide (pooled mean prevalence for AN: 1.4%, range: 0.1%–3.6% for females) and indicate an increase of the point prevalence of eating disorders over the recent years (Galmiche et al., 2019). In the Mental Health in Austrian Teenagers study a lifetime prevalence of 3.73% for any eating disorders and a prevalence of 1.44% for AN was found in 10–18‐year‐old adolescents (Wagner et al., 2017).

The treatment of AN often succeeds in temporarily restoring weight, but the starvation process in AN is often self‐perpetuating and patients have a high risk for an early relapse (Carter et al., 2012) and a chronic enduring course despite intensive treatment efforts (Wonderlich et al., 2020). Recovery of normal body weight, as one of the key elements in the treatment of AN, is often associated with gastrointestinal discomfort (GID) and abnormal fat distribution. These consequences of change can cause recurrent weight loss and could make them more prone to relapse, and may contribute to a chronic course of this illness (El Ghoch et al., 2016).

Recent studies on the gut‐brain‐interaction provide a promising basis to develop innovative interventions for patients with psychiatric illnesses (Bambury et al., 2018)*,* in particular for AN. Previous research has already identified the important link between the gut‐microbiome (MI) and the regulation of body weight (Smith et al., 2013)*,* on one hand, and psychological states, such as anxiety and depression (Pirbaglou et al., 2016)*,* on the other hand. There is growing evidence that the typical features of AN, malnutrition and long‐term unhealthy altered diet, have a profound influence on the MI (Herpertz‐Dahlmann et al., 2017; Mack et al., 2018). A recent study on adolescents with AN indicates that alterations in gut microbiota do not normalise with weight restoration (Schulz et al., 2021). Moreover, endocrinological consequences are primary or secondary amenorrhoea which occurs due to the lack of oestrogen (Neuman et al., 2015). This fact in turn leads to other problems, since difficult‐to‐treat patients are often medicated with olanzapine, which together with oestrogen have a significant impact on the MI (Maier et al., 2018; Neuman et al., 2015). Recent studies provided further evidence for a significant intestinal dysbiosis in AN, which was only partially improved with weight gain, for example, lower abundances of Bacteroidetes and carbohydrate utilising taxa as well as higher abundances of Firmicutes and Verrucobacteria. The latter two can increase intestinal wall permeability via mucin degrading and protein fermenting, which can further contribute to a ‘leaky gut’ syndrome and trigger an immune‐response (Mack et al., 2016).

Research on the gut‐brain axis is of particular relevance considering the neurological changes and neuropathological symptoms associated with AN. The morphology of the brain in patients with AN shows a change in form of a loss of volume in grey and white matter even in early stages (Seitz et al., 2016). These findings are linked to deficits in neuropsychological functioning (Seitz et al., 2015). Specifically, the MI has been previously linked to the reward system. In neuropsychological and neuroimaging studies it could be shown that patients with AN respond differently to reward and punishment in comparison to healthy controls, which may also explain their maladaptive response to hunger (DeGuzman et al., 2017). Findings from the activity based anorexia nervosa (ABA) animal model, which combines food restriction and running wheel availability confirm that animal models of AN have an striking reduction of astrocytes (Frintrop, Liesbrock, et al., 2018) and reduced cell neogenesis. Similar to phenomena in human AN weight loss, hyperactivity, amenorrhoea, hormonal disturbances, learning deficits and brain volume loss have been shown (Frintrop, Trinh, et al., 2018). Recently, intestinal dysbiosis was also confirmed in the ABA animal model, which was associated with the described brain volume and astrocyte‐marker alterations (Trinh et al., 2021).

A significant progress has been made in the field of studies on probiotic interventions. Remarkable effects on brain cell neogenesis were shown by the use of antibiotics and probiotics in mice model. While antibiotics induce a sustainable decrease in neogenesis, the treatment with probiotics leads to an increase of neogenesis (Möhle et al., 2016). As such pre‐clinical trials continue to show health benefits the administration of probiotics, sometimes also called psychobiotics, has been regarded as an option for develop innovative therapeutic intervention (Kelly et al., 2015).

As reported in the review by Markowiak and Slizewska (2017), the beneficial effects of probiotics on various diseases is known evident, particularly in the field of gastroenterology, but also in the field of allergology and infectiology, oncology and cardiology. Numerous studies have been published, particularly with regard to the selection and properties of individual probiotic cultures, their possible uses and their effects on health. Their main advantage is the effect on the development of the microbiota by ensuring a good balance between pathogens and bacteria that are necessary for the normal functioning of the organism. Probiotics can effectively inhibit the development of pathogenic bacteria such as *Clostridium perfringens*, *Campylobacter jejuni*, *Salmonella Enteritidis*, *Escherichia coli*, various types of *Shigella*, *Staphylococcus*. In addition, they increase the efficiency of the immunological system, improve the absorption of vitamins and minerals, stimulate the formation of organic and amino acids and can thus contribute to the prevention and treatment of various diseases (Markoviac & Slizewska, 2017). Specifically, probiotics interventions were tested in individuals with a variety of gastrointestinal symptoms whereby beneficial effects regarding improvements of gastrointestinal complaints were observed in about half the studies (Hungin et al., 2018).

With regards to mental health, preliminary clinical evidence suggests that the administration of probiotics could have beneficial effects in healthy individuals including significant improvements in positive affect and depressive mood and regarding significant changes in functional connectivity (e.g., in the salience network) as found in neuroimaging assessments (Bagga et al., 2018; 2019). In a sample of healthy college students, it was shown that multispecies probiotics improved panic anxiety, neurophysiological anxiety, negative affect, worry and mood regulation (Tran et al., 2019).

The effect of probiotic interventions have been also evaluated in patients with psychiatric disorders, including patients with major depression disorder, schizophrenia, bipolar disorder and Alzheimer disease (Amriani et al., 2020; Sanada et al., 2020). These studies indicate that probiotics supplementation may be effective in reducing symptoms of mental health disorders such as depression; however, the number of existing studies is still limited and definitive conclusions cannot be drawn. A systematic review on the impact of prebiotic/probiotic supplementation in children and adolescents with neuropsychiatric disorders including patients diagnosed with attention‐deficit/hyperactivity disorder and autism spectrum disorder, revealed significant improvements in the gut microbiota and behavioural changes (e.g., increased prosocial behaviour in autism spectrum disorder) (Ligezka et al., 2021).

Although profound alterations in the gut MI of patients with AN are evident (Di Lodovico et al., 2021) qualifying these patients as candidates to explore the effects of probiotics supplementation, no studies have evaluated the use of probiotics in the treatment of AN, so far. Thus, the aim of this study is to investigate the beneficial effects of a multistrain probiotics administration for 6 months in adolescent patients with AN. After treatment with probiotics, we expect alterations in the MI approaching composition of healthy‐weight individuals, improvement of gastrointestinal complaints and of depressive symptoms, a reduction of inflammatory processes and support weight gain post intervention and at 1‐year follow‐up. Probiotics may serve as a novel treatment component that minimizes the risk of relapse and a chronic course for this serious disorder of youth.

## 2 METHODS

### 2.1 Objectives and hypotheses

This study will investigate the clinical efficacy of administration of probiotics (in addition to treatment as usual, TAU) for 6 months in hospitalised adolescents with AN compared with placebo and follow them up at 6 months and 1 year. The core focus of this study is to evaluate whether probiotic supplementation support weight gain, improve eating disorder psychopathology, other psychopathology and gastro‐intestinal symptoms.

Specifically, our primary and secondary hypotheses are as follows.

#### 2.1.1 Primary hypothesis

- Patients with AN who receive a probiotic supplementation for 6 months show greater weight gain compared to patients who receive placebo.

#### 2.1.2 Secondary hypotheses

- Patients with AN who receive probiotics show a greater reduction in psychopathology which includes eating disorder pathology, depression, anxiety and obsessive‐compulsive behaviour compared to patients who receive placebo.
- Patients with AN who receive probiotics show a greater reduction in gastrointestinal complaints compared to patients who receive placebo.
- The composition of the gut MI of patients with AN who receive probiotics gets closer to the MI of healthy individuals compared to patients with AN who receive placebo.
- Patients with AN who receive probiotics show improved neuropsychological functioning (including cognitive flexibility, executive functioning, sustained attention) compared to patients who receive placebo.

Furthermore, associations of the MI with structural and functional brain‐imaging, serum (e.g., IgG, inflammatory markers, hormones), neuropsychology and psychopathology will be explored. Details regarding the acquisition and analysis of brain‐imaging data will be reported in a separate protocol paper.

In addition, the interactions of the MI and inflammation markers, gut permeability and hormones are tested to study their role in the potential anti‐inflammatory effect of probiotics in AN. Our hypotheses are that probiotics will influence the gut–brain axis in AN by an anti‐inflammatory effect mediated by the MI.

### 2.2 Overarching project

This study is part of the ERANET‐NEURON consortium ‘Microbiome Gut‐Brain interaction in Anorexia Nervosa’ (MiGBAN) which aims to investigate the gut‐brain interaction in adolescent and adult patients with AN and in animal models by means of longitudinal studies and randomized‐controlled trials. The overarching MiGBAN project is coordinated by the Department of Child and Adolescent Psychiatry, Psychosomatics and Psychotherapy, RWTH Aachen University, Germany. Another interventional study with a parallel study design will be conducted by the RWTH University of Aachen which will investigate the effect of supplementation of omega‐3 poly‐unsaturated fatty acids (PUFA) instead of probiotics in adolescents with AN.

### 2.3 Study design and recruitment

**FIGURE 1:**
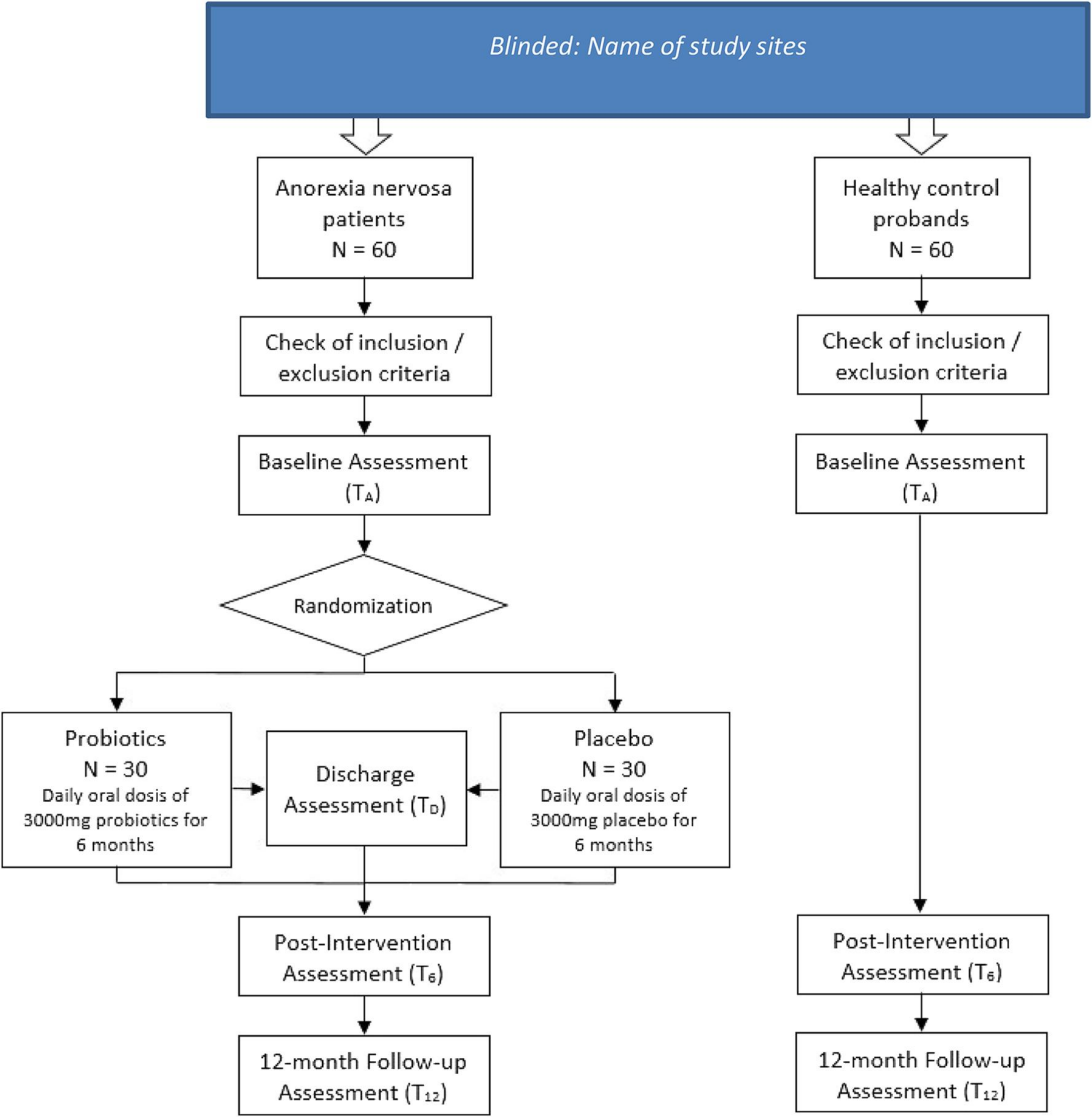
Study design of the present study. Blinded: name of study sites

The present study is a two‐centre, longitudinal, double‐blind, randomised, placebo‐controlled trial (RCT) comparing the clinical efficacy of administration of probiotics (in addition to TAU) compared with placebo in 60 patients with AN. TAU consists of psychiatric/psychotherapeutic inpatient treatment with nutritional rehabilitation aimed at the restoration of a healthy weight and starting a psychotherapeutic process (details on TAU with outcome data can be found in Mairhofer et al. [2021]). It is important to include inpatients only, as nutritional rehabilitation needs supervision and very detailed reporting of food, fluid and probiotics/placebo intake. Outcome data will be compared to 60 sex‐ and age‐matched healthy controls who will be followed for one year but do not receive any intervention. The sample of healthy controls will serve as a baseline for a general comparison of the MI and other variables of interest between patients with AN and healthy controls. The study design is summarised in Figure 1. Hospitalised patients with AN will mainly be recruited from the Medical University of Vienna (Department of Child and Adolescent Psychiatry, Eating Disorders Unit) and additionally from the Medical University of Innsbruck (Department of Child and Adolescent Psychiatry. Healthy controls will be recruited from the Vienna study site and the German cooperation/coordination site at RWTH‐Aachen University via flyers, posters and social media.

### 2.4 Inclusion and exclusion criteria

Inpatients aged 13–19 years, who are admitted to the Eating Disorders Unit at the Departments of Child and Adolescent Psychiatry, Medical University Vienna and Medical University of Innsbruck with the diagnosis of AN (307.1) or atypical AN (307.59) according to DSM‐5 will be eligible for the study. Written informed consent from participants and one legal representative, if patients are younger than 18 years, are required. Patients with comorbid disorders including organic brain disease, psychotic or bipolar disorder, substance use disorder, serious self‐injury, diabetes, those with insufficient knowledge of the German language, and those who used antibiotics during the last 6 weeks will be excluded. Further exclusion criteria are pregnancy and organic disorders with impact on the gastrointestinal tract (e.g. inflammatory bowel disease, coeliac disease). Previous operations on the gastrointestinal tract will be evaluated in the individual case. The same inclusion and exclusion criteria apply to the healthy controls; however, they have to be absent from any current diagnosed psychiatric disorder and current or lifetime eating disorder.

### 2.5 Procedure

Trial participants who are eligible will be informed about the study procedures by the research staff and written informed consent will be obtained separately from the participants and one of the legal representatives, when the patients are younger than 18 years. The participants and the legal representatives are asked whether they consent to the storage and further analyses of blood and stool samples even in case of drop out from the study. After informed consent was obtained, patients will be randomized to the probiotics or placebo conditions (see section *Randomisation*) and baseline assessment (T_A_) of outcome variables including neuropsychological measures, eating disorder related and other psychopathology, collection of blood and stool samples will be performed (see Section 2.8). As soon as baseline assessments are finished, the intervention (administration of probiotics or placebo) is stared (see Section 2.6). Assessments will be repeated at discharge (T_D_, only in patients with AN), 6 months after baseline (post‐intervention, T_6_) and at 12‐month follow‐up (T_12_).

The trial procedures were approved by the local Research Ethics Committee at the Medical University of Vienna (reference number 2226/2018; 30 April 2019) and the Ethics Committee at the Medical University of Innsbruck (reference number 1118/2019; 21 August 2019) and will be carried out in accordance with the Declaration of Helsinki. The study was registered at the German Clinical Trials Register (DRKS00017726, 30 August 2019).

### 2.6 Intervention

A total of 30 patients will receive daily oral doses of 3000 mg of a commercially available multistrain probiotics produced by Allergosan, Graz, Austria (‘OMNI‐BIOTIC Stress Repair’ including the following bacterial strains: *Lactobacillus casei* W56, *Lactobacillus acidophilus* W22, *Lactobacillus paracasei* W20, *Bifido‐bacterium lactis* W51, *Lactobacillus salivarius* W24, *Lactococcus lactis* W19, *Bifidobacterium lactis* W52, *Lactobacillus plantarum* W62 and *Bifidobacterium bifidum* W23) for a duration of 6 months (once daily). The probiotics used in this study are a commercially available dietary supplement. No side effects are known. The participants are informed that they may experience minor gastrointestinal inconvenience at the beginning (e.g., minor abdominal fullness, flatulence, minor constipating or laxative effects). 30 patients will receive placebo (also prepared by Allergosan). The ingredients of the placebo are made up of maize starch, maltodextrin, potassium chloride, magnesium sulphate and manganese sulphate. The study medication (probiotics and placebo) is a vegan product. The administration of placebo is necessary for the successful blinding of participants and the research staff.

The participants will be trained for the self‐administration of probiotics/placebo by the nursing staff, who observes the continuous administration for the time of the inpatient stay. After discharge, the patients and the parents/caregivers get a written instruction for the further administration, which is attached to the ‘medication’ box. The participants are asked to collect the daily sachets—empty or full—in the box to comprehend the number of taken and not taken probiotics. For further questions, especially for the intervention of the probiotic administration, the contact of the research team is provided in the patient letter.

### 2.7 Randomisation

Randomisation will be carried out double‐blind by the Institute of Medical Informatics and Statistics, University of Kiel (Germany). Each patient will be allocated to one of the two treatment conditions (probiotics or placebo) in a 1:1 ratio. Randomisation will be stratified for the clinical centre (Vienna and Innsbruck study site) and atypical/typical AN according to International Classification of Diseases and Related Health Problems—10th revision (ICD‐10; World Health Organization, 2009) (i.e. whether the age‐ and sex‐specific BMI percentile at admission is above or below 10). Experimental conditions will be randomized in blocks of four individuals for patients with typical AN and in blocks of two for atypical AN participants. Block randomisation is performed using the R package randomiseR (Uschner et al., 2018).

In order to ensure a proper randomisation procedure, the study medication (probiotics or placebo) is prepared for each individual participant by the same company (Allergosan) and consecutively numbered. The number does not allow any conclusion about the group (probiotic vs. placebo). The allocation list is exclusively sent to the Institute of Medical Informatics and Statistics, University of Kiel (Germany) which performs the randomisation and informs the clinical centre about the number of the study medication that should be given to the individual participant. All other parties involved, including patients, clinicians and research staff who are responsible for recruiting participants and obtaining data for the evaluation of this study have no insight into the allocation list until the blinding is removed at the end of the study (after the final patients has completed the 12‐month follow‐up assessment). Importantly, probiotics and placebo do not differ regarding packaging, smell, taste and appearance.

### 2.8 Measures and instruments

In order to evaluate the effect of probiotic administration in patients with AN, various measures are obtained at baseline, discharge, post‐intervention and 12‐month follow‐up. Assessments at discharge are omitted if the post‐intervention assessment (6 months after baseline) is scheduled 2 weeks before or one month after discharge. A complete list of all measures and instruments used in patients and healthy controls including the time points when they are assessed is provided in Table 1.

**TABLE 1:** Summary of measures and instruments used in the present study

Instrument/measure	Domain/aim	Time point^a^			
		T_A_	T_D_	T_6_	T_12_
Sociodemographic questionnaire	Key sociodemographic and clinical data	x	–	–	–
Body‐mass‐index (BMI)	Weight/weight gain	x	x	x	x
Eating disorder examination interview (EDE; Cooper & Fairburn, 1987)^b^	Eating disorder diagnosis and symptomatology	x	x	x	x
Eating disorder examination questionnaire (EDE‐Q; Hilbert et al., 2007)	Eating disorder symptomatology (self‐report)	x	x	x	x
Eating disorder inventory‐2 (EDI‐2; Garner, 1991)	Eating disorder symptomatology	x	x	x	x
MINI interview/MINI‐KID interview (Sheehan et al., 1998)	Psychiatric comorbidity	x			
Beck depression inventory‐II short version (Beck et al., 2006)	Level of depression	x	x	x	x
State‐trait anxiety inventory (state part only) (Spielberger, 2010)	Level of anxiety	x	x	x	x
Yale‐brown obsessive compulsive scale (Y‐BOCS; Goodman et al., 1989)	Obsessive compulsive thoughts and behaviours (self‐report and expert rating)	x	x	x	x
Social responsiveness scale (SRS) (Bölte & Poustka, 2007)^b^	Autistic traits (parent report)	x	‐	x	x
Gastrointestinal complaints questionnaire (Cuntz & Hiller, 1998)	Gastrointestinal complaints	x	x	x	x
Iowa gambling task (IGT; Bechara et al., 1994)	Executive functioning (decision‐making processes)	x	‐	x	x
Probabilistic reversal learning task (PRLT; Cools et al., 2009)	Cognitive flexibility	x	‐	x	x
Food and non‐food related Go/No go task (Rosval et al., 2006)	Sustained attention and response control	x	‐	x	x
UPPS‐P impulsive behaviour scale (negative urgency‐scale only) (Whiteside & Lynam, 2001)	Level of emotion‐driven impulsiveness	x	‐	x	x
BIS/BAS questionnaire (Carver & White, 1994)	Sensitivity for reward and punishment	x	‐	x	x
Wechsler intelligence test (two subtests: Matrices and vocabulary) WISC‐V (Petermann, 2017) or WAIS‐IV (Petermann, 2012)	Intelligence	‐	‐	x	‐
Stool sample	Composition of the gut‐microbiome	x	x	x	x
Food protocol	Dietary intake during 48 h prior stool sampling	x	x	x	x
Actimetre/physical activity tracker	Amount of physical activity (steps, active hours) during 48 h prior stool sampling	x	x	x	x
Serum/plasma sample	Diverse blood parameters including Hormones, gut permeability parameters (serum zonulin), systemic inflammation markers (interleukin IL‐6), hunger and satiety markers (ghrelin, α‐MSH‐reactive IgG)	x	x	x	x
(Functional) magnet resonance imaging^c^	Analysis of the underlying mechanism of the gut‐brain interaction	x	‐	x	x

^a^ Definition of assessment time points: T_A_ baseline assessment at admission, T_D_ discharge assessment (only in anorexia nervosa patients), T_6_ post‐intervention assessment (6 months after baseline), T_12_ 12‐month follow‐up assessment.

^b^ Assessed in anorexia nervosa patients only (not in healthy controls).

^c^ Assessed in 30 anorexia nervosa patients and 60 healthy controls only.

#### 2.8.1 Primary outcome measure

The primary outcome measure is defined as the body‐mass‐index (BMI) and sex‐ and age‐specific BMI percentiles based on KIGGS reference data (Robert Koch Institute, 2013). Height and weight measures are taken by trained nurses.

#### 2.8.2 Secondary outcome measures

The comprehensive test battery for the secondary outcome include in‐depth assessment of the eating disorder diagnosis and symptomatology using a gold‐standard clinical interview (Cooper & Fairburn, 1987) and self‐report instruments (Garner, 1991; Hilbert et al., 2007), and assessment of comorbid psychiatric disorders using a structured diagnostic interview (Sheehan et al., 1998) and self‐report questionnaires for depression (Beck et al., 2006), anxiety (Spielberger, 2010), obsessive compulsive thoughts and behaviours (Goodman et al., 1989) and autistic traits (Bölte & Poustka, 2007). Gastrointestinal complaints are obtained by obtaining the frequency of and distress caused by 17 gastrointestinal symptoms (e.g. flatulence, stomach ache, very hard or soft defecation, regurgitation) (Cuntz & Hiller, 1998). Moreover, a battery of neuropsychological tests are obtained including measures of decision making processes (Bechara et al., 1994), cognitive flexibility (Cools et al., 2009), sustained attention and response control (Rosval et al., 2006), level of emotion‐driven impulsiveness (Carver & White, 1994), sensitivity for reward and punishment (Carver & White, 1994) and a brief assessment of intelligence (Petermann, 2012, 2017).

Furthermore, stool samples are collected at each time point to analyse the composition of the gut MI. We also obtain the dietary intake by food protocols and the level of physical activity by using an actimeter/fitness tracker (Fitbit, Inc.) during 48 h prior to the collection of stool samples as it is known that the gut MI is influenced by the composition of the nutrients that are ingested and the amount of physical activity. We also collect information about alcohol (Engen et al., 2015) and drug consumption (Meckel et al., 2019), medication use (Bahr et al., 2015; Maier et al., 2018) including antibiotics (Arnau et al., 2020) and prebiotics (Parnell et al., 2012) during the previous six months as these factors are known to impact the gut MI. Moreover, previous infection with COVID‐19 and COVID‐19 vaccination status is obtained as their influence on the intestinal MI is still unknown; however, first evidence indicate that the MI may moderate the effectiveness of the vaccination (Lynn et al., 2021).

We also take serum/plasma samples to analyse markers of gut permeability (serum zonulin), systemic inflammation (interleukin IL‐6), hunger and satiety (ghrelin, α‐MSH‐reactive IgG) as well as hormones (e.g., LH, FSH, cortisol).

Finally, we also conduct structural (sMRI) and functional magnet resonance imaging (fMRI) to better analyse the underlying mechanism of the gut‐brain‐interaction in a subsample of patients with AN and healthy controls. Details regarding the sMRI and fMRI procedure will be published in a separate protocol paper.

### 2.9 Storing and processing of blood and stool samples

Blood serum samples will undergo centrifugation and are separated into aliquots of 500 µl and stored at −80°C. Analysis for inflammatory markers, gut permeability markers and hormones will be conducted using ELISAs.

For the collection of stool samples the patient is instructed to line the toilet bowl with toilet paper before defecation to guarantee an accurate sample collection. Further, they are instructed to put three pea‐sized amounts of stool with the spoon, which is attached to the stool tube, into the tube and then to cap the tube.

After collection, the stool samples will be sent to the Biobank, divided into three aliquots and frozen at −80°C as fast as possible (usually within 24 h). For follow‐up assessments and healthy control subjects, stool tubes are sent by post or provided at an appointment at the clinic. They are instructed to collect the stool sample at home and bring it to their appointment at the clinic. The stool sample is then stored within 48 h.

In order to analyse the MI composition frozen stool samples collected from patients and HC will be subject to microbial genomic DNA extraction. Briefly, total DNA will be extracted using the QIAamp PowerFecal DNA Kit (Qiagen), following the manufacturer's protocol. The V3‐V4 region of the 16S rRNA gene will be amplified and sequenced on an MiSeq Illumina sequencer. Raw reads will be quality checked using FastQC v0.11.6 (Andrews et al., 2015). QIIME2 will be used to process and analyse the sequence data (Bolyen et al., 2019). Paired end sequences will be denoised by using dada2 (Callahan et al., 2016), and clustered into amplicon sequences variants (ASVs) using ‘vsearch’ with an identity of 0.97 (Rognes et al., 2016). Bacterial ASVs will be annotated using q2‐feature‐classifier plugin (Bokulich et al., 2014). The ASV table will be read into R v3.2.3 for statistical investigation with the vegan package (Dixon, 2003).

Blood and stool samples are stored at BIOBANK General Hospital of Vienna and BIOBANK of the Medical University of Innsbruck. All samples will be labelled with study pseudonyms containing the respective measurement time point and dates of sampling.

### 2.10 Data management

All questionnaires and neuropsychological tests will be presented on a laptop using Inquisit software (Millisecond, version 5, 2016) in most cases. All other data collected using paper‐pencil methods or interviews will be entered into a local clinical trials data base (Open Clinica LLC).

### 2.11 Assessment of adverse events

In recent studies only mild adverse effects are described for probiotics and it is expected that patients who are randomized to the probiotic intervention will directly benefit from this treatment. In case of an adverse effect related to the administration of probiotics the physician can decide to stop the intervention. The described reactions will be documented in the case report form. In case of serious adverse effects in relation to the administration of probiotics the batch number can be singularly unblinded. In addition, all serious adverse events (e.g., death, suicidality, serious self‐injury, serious medical complications) as well as hospital re‐admission are documented regardless of their connection with the provided intervention.

### 2.12 Sample size considerations

So far, no studies on the specific effects of a probiotic intervention in patients with AN are available. A small study that investigated the effects of providing omega‐3 poly‐unsaturated fatty acids (PUFA) compared to saturated fatty acids in patients with AN found an effect in weight gain of *d* = 1.0 (Mauler et al., 2009). Meta‐analyses of probiotic interventions in depression and anxiety disorders revealed a pooled effect of *d* = 0.68 for improvements in depressive symptoms and *d* = 0.66 for improvements in anxiety symptoms compared to placebo (Huang et al., 2017). Thus, for the present study we assumed an effect size of *d* = 0.75 for the effect of probiotics compared to placebo on the change in BMI. To detect this effect using a two‐sided *t*‐test with a power of 80% at a significance level of 0.1, a sample size of 24 patients per group is needed. Considering a dropout rate until the 12‐month follow‐up of about 10%, 30 patients per group, respectively, 60 patients in total will be included in the trial. The final analysis will be performed after the complete follow‐up of all the patients. In case the final analysis will include 30 patients per group, the expected power (assuming an effect size of *d* = 0.75) will be 88%. For effect sizes of *d* = 1.0 and *d* = 0.65 as reported by previous studies mentioned above, the achieved power will be 98% and 80%, respectively.

### 2.13 Statistical analysis plan

The primary analysis will be based on the intention‐to‐treat principle (ITT), including all randomized patients irrespective of the amount of treatment actually received. A per‐protocol analysis will be performed as sensitivity analysis. In order to follow ITT principle as closely as possible, all participants will be asked to participate in the end‐of‐treatment and follow‐up assessment (at 6 months and 1 year), in particular regarding BMI measurement, even if they drop out of treatment, to minimise the amount of missing data.

We will test for a difference in the primary outcome measure (age‐adjusted BMI at 1 year follow‐up) between the probiotics and placebo arm using a mixed‐effects model repeated measures analysis with covariates study site, sex, atypical/typical AN, and BMI at admission and will include any available further measurements of BMI (in particular at discharge and month 6). As this is equivalent to a phase II trial, emphasis will be on the 95% confidence intervals of the effect size estimates; additionally, *p*‐values will be calculated.

Secondary outcomes such as eating disorder pathology and changes in the gut MI will be analysed in a similar way, additionally using measures of MI community composition and diversity measures, mostly in a descriptive way.

In addition to the randomized comparison between probiotics and placebo groups, a comparison between healthy controls and patients (in both arms) will be done to assess both differences in gut MI composition and diversity between patients with AN and healthy controls in the most acute phase of disease (at admission to inpatient treatment) and to assess the size of changes during and after treatment and weight rehabilitation in patients with AN compared to natural fluctuations of MI composition in healthy adolescents during normal development. Again, mixed‐effects models repeated measures analyses with an emphasis on descriptive reporting of confidence intervals of effect sizes will be performed.

In this clinical trial, any missing values of the primary outcome variable have to be considered to be missing not at random (MNAR). In particular patients with very poor response to the intervention and the standard multi‐modal treatment of AN that is the same in both arms, might be more likely to drop out of treatment and might be more likely not to provide data on the primary outcome (loss to follow‐up). Thus, imputation strategies or analysis methods that rely on the missing at random (MAR) assumption could be anti‐conservative. However, every effort will be made to keep missing data as low as possible. In the primary analysis of the primary endpoint, we will use all available measures of the primary outcome BMI at all time points in a mixed‐effects model repeated measures analysis.

Sensitivity analyses will be performed to investigate the potential impact of missing data, in particular, by performing a complete case analysis, a pre‐specified conservative single imputation approach and by using multiple imputations.

## 3 DISCUSSION

This is the first study investigating the administration of probiotics in adolescent patients with AN and evaluating the effects on weight gain, eating disorder pathology, neuropsychological symptoms and brain functioning. In this regard, the present study can fill a significant research gap and will provide useful insights how a therapeutic intervention directly affecting the gut MI may impact the AN symptomatology and illness course. The current research may also contribute to an improved understanding of the pathogenesis of AN by gaining better insight into the role of the gut MI and can open up new therapeutic interventions. Furthermore, this study also greatly contributes to the research on probiotics use for children and adolescents with psychiatric disorders in general, as previous studies investigating the link between the gut MI, probiotic supplementation and symptom improvement have mainly focussed on adult patients; however, none of them has studied the impact of a probiotics interventions in patients with AN yet. The strengths of the present study are the double‐blind randomized longitudinal design, the large sample size and the comparison with sex and age‐matched healthy controls. The longitudinal observation of healthy adolescents particularly allows to consider normal age‐related changes in the gut MI which will help to interpret findings obtained from the RCT in patients with AN. Furthermore, the instruments used in this study including diverse somatic, psychological and neuropsychological outcome measures provides the opportunity to not only evaluate the effects of probiotics on core features of eating disorders (BMI, eating disorder symptoms), but also on related comorbid psychopathology including depression, anxiety and compulsions as well as on neuro‐psychological features (cognitive flexibility, sensitivity to reward and punishment, impulsiveness) known to be impaired in patients with AN and known to influence progression of this disorder. If probiotics could help shift the MI composition, reduce inflammation, support weight gain and help to reduce eating disorder symptoms, its administration would be a readily applicable and easy to administer additional component of the multi‐modal AN treatment which might contribute to a better outcome of this disabling disorder.

## CONFLICT OF INTERESTS

The authors declare no conflict of interest. ‘The funders had no role in the design of the study; in the collection, analyses, or interpretation of data; in the writing of the manuscript, or in the decision to publish the results.’ All probiotics and placebos used have been bought (funded by the Austrian Science Foundation) from the company Allergosan Inc. and this company had no influence on any parts of the study.

## PATIENT CONSENT STATEMENT

Informed consent was obtained from all subjects involved in the study and their legal representatives (if patient <18 years).

## CLINICAL TRIAL REGISTRATION

This trial was registered at the German Clinical Trials Register (DRKS00017726, 30 August 2019).

## AUTHOR CONTRIBUTIONS

Conceptualisation: Andreas Karwautz, Beate Herpertz‐Dahlmann, Jochen Seitz, Serguei Fetissov and Michael Zeiler. Methodology: Michael Zeiler, Lara Keller, Astrid Dempfle, Jochen Seitz, John F. Baines, Roger Adan, Annemarie van Elburg, John F. Baines. Formal analysis: Micheal Zeiler, Astrid Dempfle. Investigation: Eva‐Maria Gröbner, Florian Ph. S. Fischmeister, Kathrin Kollndorfer, Sonja Schmelz, Andrea Schneider, Michael Zeiler, Nadia A. Andreani, Unna Danner, Nina Haid‐Stecher, Kathrin Sevecke, John F. Baines. Resources: Andreas Karwautz; data curation, Astrid Dempfle. Writing—original draft preparation: Eva‐Maria Gröbner, Michael Zeiler. Writing—review and editing: Michael Zeiler, Florian Ph. S. Fischmeister, Andreas Karwautz, Gudrun Wagner. Visualization: Michael Zeiler. Supervision: Andreas Karwautz, Florian Ph. S. Fischmeister, Michael Zeiler. Project administration: Eva‐Maria Gröbner, Michael Zeiler. Funding acquisition: Andreas Karwautz, Beate Herpertz‐Dahlmann, Jochen Seitz. All authors have read and agreed to the published version of the manuscript.

## Data Availability

As this is a study protocol paper, no original data are presented.
